# Selection on non-antigenic gene segments of seasonal influenza A virus and its impact on adaptive evolution

**DOI:** 10.1093/ve/vex034

**Published:** 2017-11-09

**Authors:** Jayna Raghwani, Robin N Thompson, Katia Koelle

**Affiliations:** 1 Department of Zoology, University of Oxford, Oxford, OX1 3SY, UK; 2 Department of Biology, Duke University, Durham, NC 27708, USA

**Keywords:** seasonal influenza A/H3N2, virus adaptation, reassortment, linkage effects

## Abstract

Most studies on seasonal influenza A/H3N2 virus adaptation have focused on the main antigenic gene, hemagglutinin. However, there is increasing evidence that the genome-wide genetic background of novel antigenic variants can influence these variants’ emergence probabilities and impact their patterns of dominance in the population. This suggests that non-antigenic genes may be important in shaping the viral evolutionary dynamics. To better understand the role of selection on non-antigenic genes in the adaptive evolution of seasonal influenza viruses, we have developed a simple population genetic model that considers a virus with one antigenic and one non-antigenic gene segment. By simulating this model under different regimes of selection and reassortment, we find that the empirical patterns of lineage turnover for the antigenic and non-antigenic gene segments are best captured when there is both limited viral coinfection and selection operating on both gene segments. In contrast, under a scenario of only neutral evolution in the non-antigenic gene segment, we see persistence of multiple lineages for long periods of time in that segment, which is not compatible with observed molecular evolutionary patterns. Further, we find that reassortment, occurring in coinfected individuals, can increase the speed of viral adaptive evolution by primarily reducing selective interference and genetic linkage effects. Together, these findings suggest that, for influenza, with six internal or non-antigenic gene segments, the evolutionary dynamics of novel antigenic variants are likely to be influenced by the genome-wide genetic background as a result of linked selection among both beneficial and deleterious mutations.

## 1. Introduction

Seasonal influenza is a major infectious disease that causes 3–5 million worldwide cases of severe illness and 250,000–500,000 deaths each year in humans (WHO 2014). Of the currently circulating flu viruses, influenza A subtype H3N2 is the predominant virus contributing to these morbidity and mortality estimates. This virus is known to rapidly evolve, particularly antigenically (Hay et al. 2001), enabling it to perpetually evade herd immunity and re-infect individuals in the population. Consequently, there has been great interest in understanding how this virus evolves antigenically, especially with respect to its main antigenic gene, hemagglutinin (HA). In particular, these investigations have focused on identifying key sites involved in viral antigenicity (Wiley et al. 1981; Wilson and Cox 1990; Bush et al. 1999; Koel et al. 2013), which has provided compelling evidence of immune-mediated selection acting upon HA.

However, the limited standing genetic diversity observed for HA has been difficult to reconcile based on recurrent positive selection alone, since the high virus mutation rate and the presence of strong diversifying selection predicts a large antigenic repertoire over time (Ferguson et al. 2003). The observed low-level genetic diversity of the HA is reflected in its spindly, ladder-like phylogeny, which indicates that only a single viral lineage persists over time. Genetic variants belonging to this persisting lineage have been characterized antigenically, indicating that every 2–8 years a major antigenic change occurs that necessitates the updating of components of the seasonal influenza vaccine (Plotkin et al. 2002; Smith et al. 2004; Koel et al. 2013). Phylodynamic models have proven to be invaluable to understanding how host immunity and viral evolution can lead to these interesting phenomena of a spindly phylogeny and a single major circulating antigenic variant dominating global infection dynamics (Ferguson et al. 2003; Koelle et al. 2006; Bedford et al. 2012; Zinder et al. 2013). Although these models differ in their specific explanations of what processes shape this restricted antigenic evolution of influenza A/H3N2, they, in general, have had to either impose strong among-strain competition for susceptible hosts (Ferguson et al. 2003; Bedford et al. 2012; Zinder et al. 2013) and/or limit the antigenic mutation rate (Koelle et al. 2006; Zinder et al. 2013). More recent work on the molecular evolution of the HA indicates that clonal interference and background selection are also important determinants of the adaptive dynamics of the HA (Illingworth and Mustonen 2012; Luksza and Lassig 2014; Koelle and Rasmussen 2015; Kim and Kim 2016).

Although it is clear that the evolution of HA is a key component of influenza A/H3N2’s adaptive evolution, the role of other gene segments, in particular those that encode internal proteins, is less well-understood. There is a growing number of studies that indicate that selection also acts on viral phenotypes beyond antibody-mediated immune escape. For example, the appearance and dominance of the CA04 antigenic lineage is attributed in part to the increased replicative fitness and virulence conferred by two amino acid substitutions in the polymerase acidic (PA) gene segment (Memoli et al. 2009). There is also evidence that cytotoxic T-lymphocyte immune pressure can exert selection pressure on influenza A virus. Specifically, recent work has shown that adaptive substitutions in the nucleoprotein (NP) gene predominantly occur at T-cell epitopes (Gong et al. 2013, 2014).

Interestingly, the genetic diversity of internal or non-antigenic genes in influenza A/H3N2 virus is also limited, although to a lesser extent than for HA (Rambaut et al. 2008). One explanation for this observation is that these gene segments are in strong linkage with HA, which means that any evolutionary force that reduces genetic diversity of the HA (e.g. selective sweeps and genetic bottlenecks) will also similarly impact the rest of the virus genome. However, whole-genome analyses of seasonal influenza A viruses indicate that reassortment is relatively frequent, with each gene segment having somewhat of a distinctive evolutionary history (Holmes et al. 2005; Nelson et al. 2008b; Rambaut et al. 2008; Westgeest et al. 2014; Maljkovic Berry et al. 2016). Estimated differences in the times to most recent common ancestor (TMRCA) across the genome can also exceed 6 years (Rambaut et al. 2008), which is inconsistent with strong linkage effects solely shaping the genetic diversity patterns of this virus. An alternative explanation for the limited genetic diversity of non-antigenic gene segments is selection specifically acting on these segments. To date, however, there has been very little consideration of the extent to which selection on these non-antigenic segments contributes to shaping their own evolutionary dynamics. Furthermore, through linkage effects, there is the possibility that selection on the non-antigenic gene segments could also shape the evolutionary dynamics of the antigenic gene segments in influenza A virus.

Here, we evaluate the importance of selection on non-antigenic gene segments in the adaptive evolution of seasonal influenza A/H3N2 by analyzing the evolutionary dynamics of the viral genome and using a population genetic model to determine the critical processes that can reproduce features of these observed evolutionary dynamics. The main questions we address are whether selection on non-antigenic gene segments impacts the evolutionary dynamics of the non-antigenic gene segments themselves, and through linkage effects, the antigenic gene segments. Instead of examining the complexity of eight distinct gene segments, we simplify our model by considering a virus that contains only two gene segments, corresponding to one antigenic gene (e.g. HA) and one non-antigenic gene (e.g. PA). By simulating the model such that lineages can be traced back in time, we examine the patterns of genetic diversity of the virus across different assumptions of selection and reassortment. We find that selective effects on both gene segments and limited reassortment (via limited coinfection rates) are necessary to capture the key TMRCA patterns of the influenza A/H3N2 virus genome. Furthermore, we find that the rate of adaptive evolution of the virus increases under this evolutionary regime, which is predominantly a result of reassortment reducing the interference effects acting upon the non-antigenic gene segment.

## 2. Materials and methods

### 2.1 Evolutionary dynamics of seasonal influenza A/H3N2 virus genome

To characterize the evolutionary dynamics of influenza A/H3N2, we used a published global whole-genome dataset of viruses sampled from 1977 to 2009 (*n *= 676) (Bhatt et al. 2011). Time-scaled trees were estimated with BEAST v1.8 (Drummond et al. 2012) by employing a relaxed uncorrelated log-normal distributed molecular clock (Drummond et al. 2006), a codon-structured nucleotide substitutional model (Shapiro et al. 2006), and a Bayesian Skygrid coalescent prior (Gill et al. 2013). Two independent chains of 200 million steps were executed for each of the eight gene segments to ensure that adequate mixing and stationarity had been achieved. The posterior tree distribution for each segment was further examined with Posterior Analysis of Coalescent Trees (PACT) (Bedford, 2015), which infers the TMRCA across the entire evolutionary history at regular intervals. To quantify and visualize patterns of genetic diversity in each segment, mean TMRCAs over time were plotted using the R package ggplot2 (Wickham 2009) and genealogical trees were plotted with ggtree (Yu et al. 2017).

### 2.2 Phylodynamic model of infection and coinfection

To explore the evolutionary processes underlying the empirical patterns of TMRCA observed for the influenza A/H3N2 virus genome, we formulated a simple population genetic model with a constant number of *N* = 1,000 infected individuals. In the model, individuals were either infected with a single virus (*I*_s_) or coinfected with two viruses (*I*_co_). We did not consider coinfection with more than two viruses. The virus genome consisted of one antigenic segment and one non-antigenic segment.

**Figure 1.**
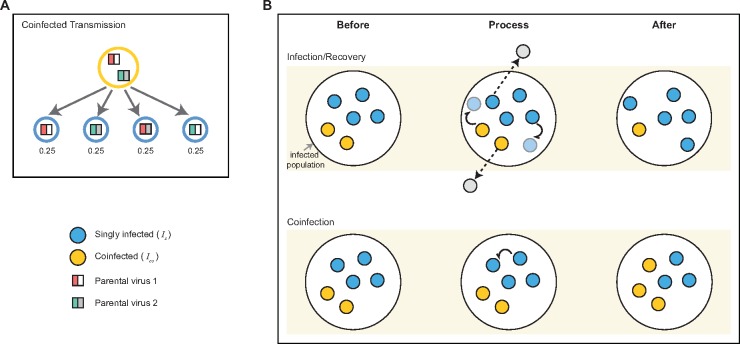
Schematic of the events in the population genetic model. (A) Infection transmission by a coinfected individual. When a coinfected individual transmits, each gene segment is randomly chosen from the two viral strains present in that individual. Consequently, there is an equal probability of transmitting a non-reassortant strain (two strains on the left) as there is of transmitting a reassortant strain (two strains on the right). (B) Schematic of the main events: infection/recovery and coinfection. Infection and recovery are coupled such that the infected population remains constant. Upon infection (indicated by curly arrows), singly infected individuals (blue circles) and coinfected individuals (yellow circles) generate new singly infected individuals. Recovery of singly infected and coinfected individuals removes them from the population (denoted by dashed arrows). Here, two infection/recovery events are shown that occur in the same time step. Coinfection events occur when a singly infected individual infects another singly infected individual. This results in a new coinfected individual in the infected population, carrying two viral strains. Coinfection events result in an increase in the number of coinfected individuals in the population and a matched decrease in the number of singly infected individuals.

We simulated the infected population of hosts over time using a modified Moran model. Specifically, we allowed for two types of infection events: ‘infection/recovery’ events and coinfection events. When an ‘infection/recovery’ event occurred, an infected individual (*I*_s_ or *I*_co_) was chosen to generate a new singly infected individual. If it was a coinfected individual generating the new infection, that individual transmitted either of the viruses he was infected with or a reassortant virus (Fig. 1A). We assumed an equal probability of each viral gene segment being transmitted, such that a reassortant strain was transmitted 50 per cent of the time. At the same time as the generation of the new infection occurred, recovery of a randomly chosen infected individual (*I*_s_ or *I*_co_) also occurred. If a coinfected individual was chosen to recover, he cleared both infecting viral strains. Because infection events were always offset by recovery events, as is traditional in Moran models where a ‘birth’ is always offset by a ‘death’, the total number of infected individuals in the population remained constant (Fig. 1B). Infection/recovery events occurred at a rate of α = 0.25 per capita per day, reflecting a typical duration of influenza infection of ∼4 days (Carrat et al. 2008).

Coinfection events were marked by singly infected individuals infecting other singly infected individuals (Fig. 1B). Coinfection events occurred from singly infected individuals at a per capita rate of β = 0.0125 per day. With α = 0.25 and β = 0.0125 per capita per day, this corresponds to a coinfection level of ∼5 per cent of the total infected population at equilibrium ( Supplementary Text S1). Ascertaining an empirical coinfection rate for influenza A/H3N2 viruses in general, or at the within-subtype level, is very difficult, since the low circulating viral diversity is likely to limit our ability to distinguish between independent infecting viral strains. Nevertheless, the number of influenza coinfections can be estimated when viral strains involved belong to either different subtypes or types (e.g. A/H3N2 and A/H1N1 or influenza A and B viruses, respectively) (Goka et al. 2013; Perez-Garcia et al. 2016). These types of coinfection have been known to occur between 1 and 2 per cent in sampled influenza A virus infections (Goka et al. 2013; Perez-Garcia et al. 2016; Poon et al. 2016). We set the level of coinfection in our model slightly higher than these empirical estimates, at ∼5 per cent, to reflect that these empirical estimates between different subtypes or types are likely underestimates.

### 2.3 Evolution of the antigenic and non-antigenic gene segments

We let mutations occur at transmission events, which consist of both ‘infection’ and ‘coinfection’ events. We let the number of new mutations present in the transmitting virus be Poisson-distributed with mean *U* = 0.1, with each mutation being equally likely to land on the antigenic or the non-antigenic gene segment. We allow the distribution of mutational fitness effects to differ between the two gene segments. Specifically, we assume that 30 per cent of mutations are beneficial and 70 per cent of mutations are deleterious on the antigenic gene segment. On the non-antigenic gene segment, we assume that 5 per cent of mutations are beneficial, 30 per cent of mutations are deleterious, and the remaining 65 per cent of mutations are neutral. A higher proportion of beneficial mutations are assumed in the antigenic gene segment to capture the selective advantage that antigenic mutations are likely to have through evasion of herd immunity. The non-antigenic gene segment is assumed to have a greater proportion of neutral mutations to reflect the observation that internal genes undergo greater neutral evolution than external genes (Bhatt et al. 2011). In line with experimentally determined distributions of fitness effects in a fast-evolving RNA virus (Sanjuan et al. 2004), we assume that the fitness effects for beneficial mutations are generally smaller than those for deleterious mutations. We specifically let the fitness effects of beneficial mutations be exponentially distributed with a mean selective advantage of 0.03 and the fitness effects of deleterious mutations be exponentially distributed with a mean selective disadvantage of 0.09. We do not consider lethal mutations.

Viral fitness is calculated by multiplying fitness values at each site across the genome. Multinomial sampling based on viral fitness is applied at each transmission event to determine which individual will infect (or coinfect) next. For coinfected individuals, we initially determine which virus is transmitted from the two infecting parental viral strains (Fig. 1A) and compute the viral fitness accordingly.

### 2.4 Tracking lineages over time

The model is implemented in Java using a Gillespie τ-leap algorithm (Gillespie 2001) for computational efficiency with a time step τ = 0.25 days. Starting from an equilibrium number of singly infected and coinfected individuals ( Supplementary Text S1), we run each simulation for 60 years, analyzing results only from the last 20 years.

To be able to infer the genealogical history of the viral population, we track in our model who-infected-whom at the level of infected individuals and for each gene segment. A random annual sample of 100 singly infected individuals is used to infer the TMRCA of each gene segment at yearly intervals. Viral gene genealogies are reconstructed from the last 20 years of simulation using a random sample of 300 singly infected individuals from over those 20 years. The tracked infection histories are used to determine the first ‘coalescent’ event, which corresponds to finding the two sampled individuals that shared the most recent common ancestor for a given gene segment. Specifically, this process involves tracing back the transmission events from the sampled infections, and establishing the parental virus in common with the most recent transmission time. This procedure is repeated until all sampled and ancestral lineages reach the parental viral infection that represents the most recent common ancestor of the entire sample.

## 3. Results

### 3.1 Genealogical diversity of seasonal influenza A/H3N2 virus

**Figure 2.**
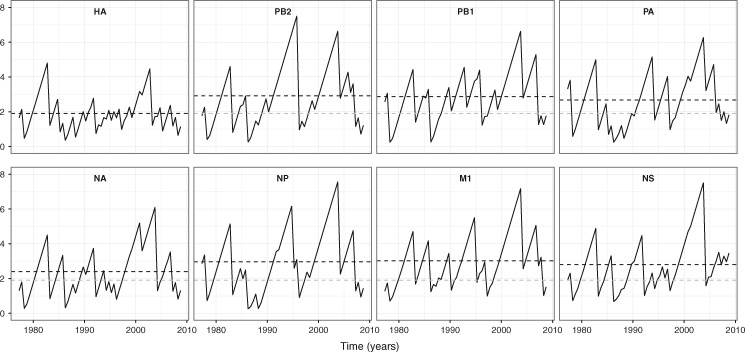
TMRCA through time plots for individual seasonal influenza A/H3N2 gene segments. The mean TMRCA over time is estimated from a posterior tree distribution for each gene segment at 6-month intervals. The black dashed lines indicate the overall mean TMRCA for the focal gene segment in each subplot. The gray dashed lines in the non-HA gene segment subplots show the overall mean TMRCA for the HA.

Figure 2 shows how the genealogical diversity of seasonal influenza A/H3N2 varies over time for each gene segment. We observe that the mean TMRCA of the HA gene segment (1.90 years) is 0.5–1.1 years younger than the other gene segments, indicating that HA experiences the fastest lineage turnover in the virus genome. The maximum TMRCA for this gene segment also does not exceed 5 years. These TMRCA patterns reflect that the HA gene genealogy has a single viral lineage dominating over time (Supplementary Fig. S1). Neuramindase (NA), the other antigenic gene, is found to have the second lowest mean TMRCA (2.4 years), indicative of slightly longer lineage persistence than HA (Supplementary Fig. S1). The non-antigenic gene segments of A/H3N2 are marked by larger mean TMRCAs and by more extensive variation in genealogical diversity over time relative to the HA gene segment (and to a lesser extent relative to the NA gene segment). This indicates that the genealogies of non-antigenic gene segments often have multiple lineages that co-exist for significant periods of time, for example up to ∼7 years in M1 (Supplementary Fig. S1). Together, these observations are compatible with positive selection acting predominantly upon the antigenic genes, most notably the HA.

### 3.2 Evolutionary dynamics when only the antigenic gene segment is under selection

**Figure 3.**
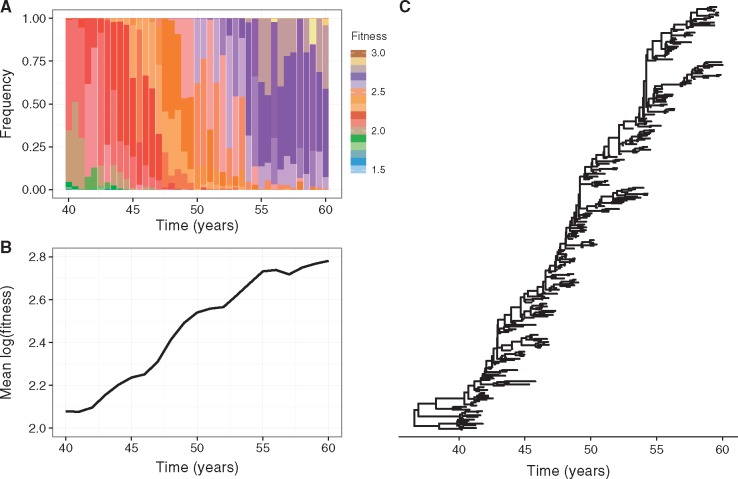
Adaptive evolution in the absence of coinfection and when selection acts only on the antigenic gene segment. Results are presented from a representative simulation. (A) Fitness variation in the viral population over time. (B) Mean (log) fitness of the virus population over time. (C) Antigenic gene genealogy reconstructed from model simulation by sampling 300 singly infected individuals over 20 years, following a burn-in of 40 years.

To better understand the patterns of genealogical diversity of the influenza A/H3N2 virus genome, we first simulated the described model under the assumption that the adaptive evolution of the virus is restricted to the antigenic gene segment, with all mutations on the non-antigenic gene segment assumed to be neutral. Further, coinfection was not permitted (β = 0). Figure 3 shows results from a representative simulation. Viruses with higher fitness constantly emerge and become dominant in the population over time (Fig. 3A). At any given time, significant fitness variation is present in the population, with lower fitness viruses able to persist in the population over extended periods (Fig. 3A).

Interestingly, the simulated viral population’s fitness appears to evolve in a punctuated manner, as indicated by the change in mean (log) population fitness over time (Fig. 3B). This result is consistent with population genetic theory that has found that beneficial mutations have to be characteristically large to be able to fix in populations undergoing background selection (Peck 1994; Barton 1995; Johnson and Barton 2002; Schiffels et al. 2011). Our simulations are capable of reproducing HA’s characteristic spindly phylogeny (Fig. 3C) and support findings from previous studies (Strelkowa and Lassig 2012; Koelle and Rasmussen 2015; Kim and Kim 2016) that have argued that HA’s spindly phylogeny occurs in an evolutionary regime where clonal interference and background selection are present.

**Figure 4.**
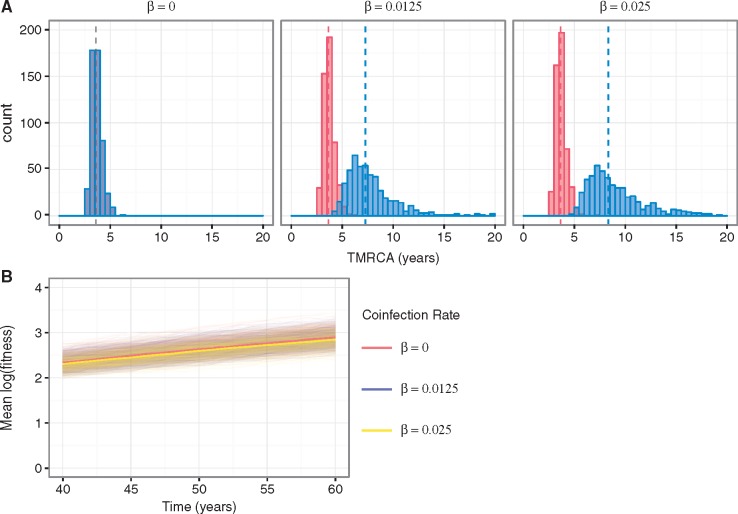
Genealogical diversity and the rate of adaptive evolution at different levels of coinfection when selection acts only on the antigenic gene segment. (A) Distribution of mean TMRCAs of the antigenic (red) and non-antigenic (blue) gene segments, obtained from 500 independent simulations for each model parameterization considered and calculated from over the last 20 years of each simulation. Three model parameterizations were considered, corresponding to different levels of coinfection: 0 per cent (β = 0), 5 per cent (β = 0.0125), and 9 per cent (β = 0.025). The dashed lines show the mean of the distributions. (B) Mean (log) fitness of the virus population over time under the three coinfection levels considered.

Under the model with both positive and negative fitness effects on only the antigenic gene segment, no significant changes in the mean TMRCA of the antigenic gene segment occur with increasing levels of coinfection (Fig. 4A). This is expected, as the extent of genetic linkage between the antigenic and non-antigenic gene segment should not impact the evolutionary dynamics of the antigenic gene segment under this scenario of no selection on the non-antigenic gene segment. In contrast, the mean TMRCA of the non-antigenic gene segment becomes notably greater as the rate of coinfection increases (Fig. 4A). This is because reassortment reduces the hitchhiking of the neutrally evolving non-antigenic gene segment with the non-neutrally evolving antigenic gene segment. As a consequence, the non-antigenic gene segment is able to explore more genetic backgrounds, which leads to an increase in its genetic diversity. Expectedly, at the level of the viral population, the rate of adaptive evolution is unaffected by the level of coinfection (Fig. 4B).

### 3.3 Reassortment increases the rate of adaptive evolution when a non-antigenic gene segment is under selection

**Figure 5.**
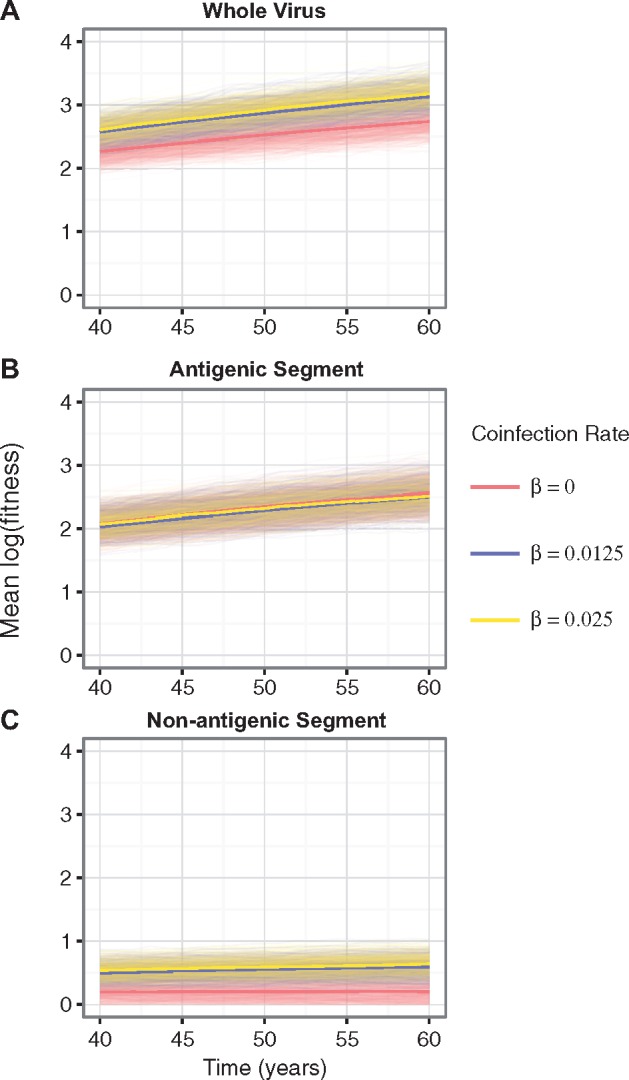
Adaptive evolution of the virus at varying levels of coinfection when both gene segments undergo selection. The mean (log) population fitness are shown for (A) the whole virus, (B) the antigenic gene segment, and (C) the non-antigenic gene segment for three different coinfection rates (β = 0, 0.0125, and 0.025 per day). Five hundred simulations were run at each of the three coinfection levels. Solid lines show the means of the simulations’ mean (log) population fitness levels.

Next, we examined the behavior of the model when selection occurs on both gene segments. First, we looked at the changes in mean population fitness and fitness variation over time under increasing coinfection rates (β = 0, 0.0125, and 0.025 per day), for the whole virus (Fig. 5A) and the antigenic (Fig. 5B) and the non-antigenic (Fig. 5C) gene segments alone. The rate of overall virus adaptation is significantly greater in the presence of coinfection than when it is absent (Fig. 5A). This phenomenon appears to be primarily driven by the non-antigenic gene segment, which also experiences a notably higher rate of adaptive evolution when coinfection occurs in the population (Fig. 5C). In contrast, the difference in the rate of adaptive evolution of the antigenic gene segment in the absence versus in the presence of coinfection appears to be only slight (Fig. 5B). Our results do not show significant differences between the β = 0.0125 and the β = 0.025 simulations, indicating that low coinfection levels of ∼5 per cent seem to be sufficient for reducing genetic linkage to the extent that is necessary to allow the viral population to rapidly adapt.

Together, these results indicate that when coinfection is absent the non-antigenic gene segment experiences greater selective interference (both among beneficial and deleterious mutations) and genetic hitchhiking than the antigenic gene segment, for the simple reason that, given our model parameterization, there are significantly more mutations with selective effects on the latter. In other words, when there is strong linkage between the two segments, selection on the antigenic gene segment will have a larger impact on impeding the adaptive dynamics of the non-antigenic gene segment than the non-antigenic gene segment will have on impeding the adaptive dynamics of the antigenic gene segment. As a consequence, while reassortment is expected to reduce linkage effects between both gene segments, larger gains in fitness are more likely for the non-antigenic gene segment under our model parameterization.

**Figure 6.**
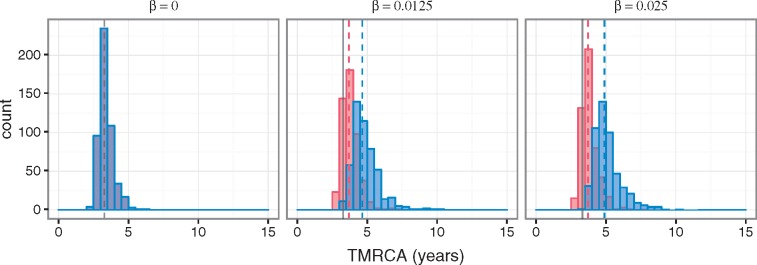
Genealogical diversity of the virus at varying levels of coinfection when selection acts on both gene segments. Distribution of mean TMRCAs of the antigenic (red) and non-antigenic (blue) gene segments, obtained from 500 independent simulations for each model parameterization considered and calculated from over the last 20 years of each simulation. Three model parameterizations were considered, corresponding to different levels of coinfection: 0 per cent (β = 0), 5 per cent (β = 0.0125), and 9 per cent (β = 0.025). The solid gray lines in the subplots indicate the mean of the simulation’s TMRCA of the antigenic gene segment in the absence of coinfection.

Coinfection also has an impact on the mean TMRCAs of both gene segments when both gene segments are targets of selection (Fig. 6). Although this pattern is much more discernible for the non-antigenic gene segment, it does indicate that the evolutionary dynamics of the antigenic gene segment are influenced to some degree by linkage effects from the non-antigenic gene segment. The larger increase in the mean TMRCA for the non-antigenic gene segment between the no coinfection and coinfection scenarios is consistent with the non-antigenic gene segment experiencing comparatively greater linkage effects than the antigenic gene segment.

### 3.4 Empirical genealogical patterns are compatible with selection on both antigenic and non-antigenic gene segments and limited reassortment

**Figure 7.**
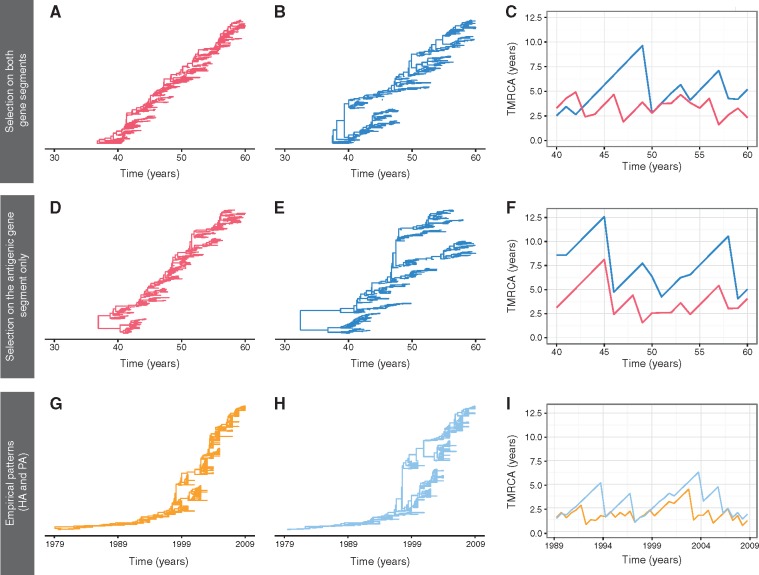
Gene genealogies and TMRCA dynamics from simulations and inferred from influenza virus gene sequences. (A–C) Representative gene genealogies and TMRCA dynamics obtained from a model simulation with selection occurring on both gene segments and a limited coinfection rate (β = 0.0125 per day). (D–F) Representative gene genealogies and TMRCA dynamics obtained from a model simulation with selection occurring on only the antigenic gene segment and a limited coinfection rate (β = 0.0125 per day). Panels A and D depict the gene genealogies of the antigenic gene segment. Panels B and E depict the gene genealogies of the non-antigenic gene segment. Panels C and F show the TMRCA dynamics of the antigenic (red) and non-antigenic (blue) gene segments over time. (G–I) Inferred influenza A/H3N2 MCC (maximum clade credibility) phylogenies for the antigenic HA gene segment and the non-antigenic PA gene segment, along with their TMRCA dynamics. The TMRCA dynamics for HA and PA are shown in panel I in orange and light blue lines, respectively. Supplementary Figure S3 shows analogous TMRCA dynamics for all six internal gene segments relative to the HA gene segment.

Figure 7 shows representative simulations of gene genealogies for the antigenic and non-antigenic gene segments under a model parameterization with a limited coinfection rate (β = 0.0125), selection acting on the antigenic gene segment, and either no selection or selection occurring on the non-antigenic gene segment. The simulated genealogies of the antigenic gene segment exhibit a single lineage persisting over time, regardless of whether the non-antigenic gene segment is evolving selectively (Fig. 7A) or neutrally (Fig. 7D). In contrast, quite different gene genealogies are observed for the non-antigenic gene segment under these two distinct scenarios (Fig. 7B and E). Specifically, the non-antigenic gene segment has significantly lower genealogical diversity when it is under selection (Fig. 7B) compared with when it is not (Fig. 7E). In both cases, however, multiple co-circulating lineages are observed more frequently in the non-antigenic gene segment than in the antigenic gene segment, indicative of slower population turnover in the non-antigenic gene segment.

When comparing TMRCA patterns between the antigenic and the non-antigenic gene segments, it is notable that when the non-antigenic gene segment evolves neutrally, the common ancestor of the non-antigenic gene segment is consistently older than that of the antigenic gene segment (Fig. 7F). Although the common ancestor of the non-antigenic gene segment is still most of the time older than that of the antigenic gene segment in simulations with selection occurring on both segments (Fig. 7C), the TMRCAs of the two gene segments occasionally coincide. This likely indicates a shared common ancestor, perhaps as a result of a genome-wide selective sweep.

To look more closely at this finding, we calculated the difference between the two gene segments’ TMRCAs over time for both sets of simulated data. Plotting these differences yielded a higher density around zero years of difference in the TMRCA for the simulation with selection on both gene segments compared with the one with selection on only the antigenic gene segment (Supplementary Fig. S2). This suggests that genome-wide selective sweeps are more likely to occur when selection occurs on both gene segments rather than on the antigenic gene segment alone.

A visual comparison between the empirical TMRCA dynamics and the simulated ones indicates that the model with selection occurring on both gene segments can more effectively reproduce TMRCA patterns observed in the empirical dynamics compared with the model with selection occurring on only the antigenic gene segment (compare Fig. 7C vs F with Fig. 7I and Supplementary Fig. S3). More concretely, similar to the simulation with selection occurring on both gene segments, the empirical TMRCA dynamics show evidence for the occasional coinciding of antigenic and non-antigenic TMRCAs, when HA is taken to be the antigenic gene segment and either PA or another internal gene segment is taken to be the non-antigenic gene segment (Fig. 7I, Supplementary Fig. S3). The model with selection occurring on both gene segments further yields TMRCA differences that are statistically more similar to those empirically observed than the model with selection occurring on only the antigenic gene segment (Supplementary Fig. S4).

### 3.5 Sensitivity of results to model parameters

#### 3.5.1 Infected population size

Although it is well-established that human influenza A/H3N2 virus has a strong seasonal transmission pattern in some populations, we decided to model a constant infected population. This decision was motivated largely by undertaking a simple and standard approach to examine the patterns of viral diversity due to selection, mutation, and reassortment alone. Given that regions with low-level, constant disease transmission (e.g. the tropics) frequently seed seasonal outbreaks in temperate locales (Rambaut et al. 2008; Russell et al. 2008; Bahl et al. 2011; Lemey et al. 2014), the effective population size of global influenza A/H3N2 viruses is also likely to be much smaller than the total population size and it may be more constant over time than would be expected by considering flu dynamics in temperate regions. To determine the sensitivity of our results to our model’s parameters, we therefore only considered the effects that different population sizes would have on the viral evolutionary dynamics (Supplementary Fig. S5). Specifically, we ran 100 simulations at each of three population sizes (*N *=* *1,000, 5,000, and * *10,000), under the model parameterization with both gene segments undergoing selection. Notably, similar TMRCA patterns were observed regardless of population size, with the antigenic gene segment typically having a smaller TMRCA compared with the non-antigenic gene segment (Supplementary Fig. S5). However, and as expected from coalescent theory, simulations run with larger population sizes had larger TMRCAs for both gene segments, indicating greater lineage persistence in the population. At larger population sizes, we can, however, recover lower TMRCAs when we increase the mean effect size of mutations (results not shown), which acts to decrease the effective population size of the viral population.

#### 3.5.2 Mutation and coinfection rates

As it is difficult to parameterize the per-genome, per transmission mutation rate *U* in our model on empirical estimates of influenza virus per-site, per replication cycle mutation rate, we considered how changes in *U* would affect model simulations. Varying *U* between 0.05 and 0.2 did not affect our general conclusion that the antigenic gene segment generally had a smaller TMRCA than the non-antigenic gene segment (Supplementary Fig. S6). At higher mutation rates, *U*, mean TMRCAs for the non-antigenic gene segment were appreciably smaller and mean TMRCAs for the antigenic gene segment were only slightly smaller (Supplementary Fig. S6), resulting in a smaller difference in the mean TMRCAs between the two gene segments.

We also looked at the sensitivity of the simulation results to the coinfection rate by varying β from 0.0025 to 0.25 per day (Supplementary Fig. S7). At the lowest coinfection rate of β = 0.0025 per day (where coinfection levels were ∼1%), the TMRCAs of the two gene segments were found to be very similar (Supplementary Fig. S7: mean difference in TMRCA is <0.5 years). Although this level of coinfection corresponds well with empirical estimates (Goka et al. 2013; Perez-Garcia et al. 2016; Poon et al. 2016), the difference in TMRCAs between antigenic and non-antigenic gene segments is not consistent with observed evolutionary dynamics (Fig. 2). Higher coinfection rates of β = 0.0125 per day (where coinfection levels were ∼5%) and β = 0.25 per day (where coinfection levels were ∼50%), yielded simulation results that were more consistent with observed evolutionary patterns in their TMRCA dynamics. A nominal amount of reassortment or more therefore appears to be necessary for observed empirical patterns to be reproduced.

#### 3.5.3 Distribution of fitness effects

To better understand the impact of our chosen distribution of fitness effects on our findings, we also considered an alternative distribution of fitness effects. This alternative distribution emulates some of the recent empirical findings from Visher et al. 2016. Specifically, we assumed that in the non-antigenic gene segment all mutations were deleterious. This reflects the empirical observation from Visher et al. 2016 that the overwhelming majority of non-lethal mutations in internal gene segments are deleterious. We further assumed that in the antigenic gene segment, 30 per cent of mutations were beneficial while the remaining 70 per cent of mutations were deleterious. This deviates from Visher et al. 2016 results in that they also found that the overwhelming majority of non-lethal mutations were deleterious in the antigenic gene segments (HA and NA). However, their quantification of fitness effects did not take into consideration the beneficial effect that mutations occurring on antigenic gene segments could have for viral immune escape. Since immune escape is generally accepted to be the dominant process driving adaptive evolution in influenza A virus, we assumed that a significant proportion of mutations in the antigenic gene segment would be beneficial to viral fitness. Beneficial and deleterious mutations were drawn from exponential distributions with means 0.3 and 0.03, respectively. These means differ from those quantified in Visher et al. 2016 but these means are not directly comparable since our model considers transmission fitness rather than fitness at the level of cellular replication.

Supplementary Figure S9 shows simulated evolutionary dynamics under the alternative distribution of fitness effects for various rates of coinfection (β = 0, 0.0125, and 0.025 per day). As in our original simulations (Fig. 5), reassortment increased the rate of adaptive evolution of the virus as whole, albeit only slightly (Supplementary Fig. S9A). The rate of adaptation of the antigenic gene segment was not appreciably affected by reassortment (Supplementary Fig. S9B), again consistent with our original findings. Finally, and again consistent with our original findings, reassortment enabled the non-antigenic gene segment to explore more advantageous genetic backgrounds and this segment became less subject to genetic hitchhiking with the antigenic gene segment (Supplementary Fig. S9C). As a result, the mean (log) fitness of the non-antigenic gene segment was higher in the models with reassortment than in the model with its absence. Over time, however, regardless of the coinfection rate, the mean (log) fitness of the non-antigenic gene segment declined. This decline makes intuitive sense since all mutations on this gene segment are assumed to be deleterious, and Muller’s ratchet will occur in this population. A slower fitness decline in non-antigenic gene segment is consistent with the effect that recombination and reassortment are known to play in slowing down Muller’s ratchet (Gordo and Campos 2008). Although our results are sensitive to whether reassortment is allowed to occur they are relatively insensitive to the level of reassortment being assumed. As long as reassortment occurs at a nominal level or higher, the decline in the fitness of the non-antigenic gene segment will be slowed.

We further examined the evolutionary dynamics of the virus when the distributions of fitness effects for both gene segments were identical, with each of the two gene segments having a distribution of fitness effects as the antigenic gene segment in the original parameterization (see Section 2). Under this parameterization, a significant increase in the rate of viral adaptation was again observed when coinfection occurred (Supplementary Fig. S10A). However, and unlike in our previous results, increases in the level of coinfection did not increase the TMRCAs of the non-antigenic gene segment relative to the antigenic gene segment, although, as expected, coinfection increased the mean TMRCAs of both gene segments (Supplementary Fig. S10B). These findings indicate that reassortment reduces selective interference among gene segments, and thereby enables fitness gains for the virus, as long as there is selection acting on both gene segments. However, selection occurring equally on both gene segments cannot reproduce the observed empirical TMRCA patterns. Specifically, this evolutionary regime cannot reproduce the observation that TMRCAs are generally larger in non-antigenic gene segments compared with HA (Supplementary Fig. S3).

## 4. Discussion

We have developed a simple population genetic model to examine the role that non-antigenic gene segments may play in the adaptive evolution of seasonal influenza A viruses. In contrast to previous phylodynamic and predictive models of influenza evolution, which have exclusively focused on the viral HA (Koelle et al. 2006; Bedford et al. 2011; Strelkowa and Lassig 2012; Zinder et al. 2013; Luksza and Lassig 2014), our model allows us to evaluate the importance of selection on non-antigenic gene segments and intrasubtypic reassortment to the molecular and adaptive evolutionary dynamics of the virus genome. We find that empirical patterns of genetic diversity in the internal gene segments and the differences in the TMRCAs between these internal gene segments and the viral HA are principally captured by a model with selection occurring on both antigenic and non-antigenic gene segments and reassortment occurring at a nominal level or higher. Furthermore, while our results indicate that selection on the non-antigenic gene segment can slightly influence the evolutionary dynamics of the antigenic gene segment, reassortment increases the rate of viral adaptation in our model primarily by decreasing selective interference acting upon the non-antigenic gene segment (rather than upon the antigenic gene segment). These findings are consistent with classical population genetic studies that have found that sex can be an evolutionary advantage in the presence of deleterious mutations and linkage effects (Hill and Robertson 1966; Colegrave 2002; Gordo and Campos 2008).

Given that only two viral segments are modeled in this study, it would be interesting to see if these results still hold when additional non-antigenic gene segments are considered. One prediction is that since linkage effects are expected to increase with additional gene segments, we are more likely to see the cumulative effect of selection acting on the non-antigenic segments on the antigenic gene segment. Consequently, in light of this hypothesis, our finding that non-antigenic gene segments have minimal impact on the rate of adaptive evolution in the antigenic gene segment is likely to be overly conservative.

Since the coinfection level assumed in our model is ∼5 per cent and each coinfected individual has a 50 per cent chance of transmitting a new reassortant strain, around 2.5 per cent of the infected population is expected to carry a first-generation reassortant virus. With eight gene segments, the chance that a coinfected individual transmits a new reassortant strain would be much higher, at nearly 100 per cent, such that around 5 per cent of the infected population would be expected to carry a first-generation reassortant virus. Interestingly, this low-level reassortment is consistent with an estimated frequency of reassortment events observed among sampled virus genomes over time, at around 3.35 per cent (Maljkovic Berry et al. 2016). This observation, in part, could be explained by the likelihood of coinfection, and thus reassortment, being reduced as result of herd immunity—that is viral infections with novel antigenic variants are less likely to occur in individuals that have been previously infected with older antigenic variants. Further evidence that intrasubtypic reassortment is restricted at the between-host level comes from a recent finding that even at the within-host scale the effective reassortment rate is very limited (Sobel Leonard et al. 2017). These observations indicate that the difference in the TMRCA across the seasonal influenza A virus genome is likely to arise from low-level reassortment in the virus population. Importantly, this has strong implications for the adaptive evolution of the virus, since it suggests that selective interference *among* gene segments, rather than only *within* gene segments (Illingworth and Mustonen 2012), has the potential to influence the fate of both beneficial and deleterious mutations in the genome.

Although reassortment is notoriously associated with pandemic influenza (Morens et al. 2009), there are several historical events in both seasonal influenza A/H3N2 and in seasonal influenza A/H1N1 where intrasubtypic reassortment has been implicated in antigenic cluster transitions (Holmes et al. 2005; Nelson et al. 2008a; Memoli et al. 2009). Furthermore, given that these instances are often associated with greater disease severity and incidence, akin to pandemic influenza, it also indicates that intrasubtypic reassortment can facilitate significant improvements in viral fitness. This is consistent with our findings that reassortment can increase the rate of virus adaptive evolution by reducing selective interference effects across the genome.

In our model, we simulated viral evolution by introducing new mutations with fitness effects sampled from a distribution, with fitness variation between viruses (and genetic drift) giving rise to changes in their frequencies. New mutations were assumed to carry fitness costs or benefits that were constant in time. Although this may be the case for many mutations, other mutations, most notably antigenic mutations, will more likely have fitness effects that are time-varying due to frequency-dependent selection. An alternative approach to incorporating the effect of antigenic mutations would be to explicitly model this frequency-dependent selection process. This type of dynamic has been previously modeled by Strelkowa and Lassig 2012, where the authors let mutations at epitope sites in the viral HA have time-varying fitness effects. Under this evolutionary regime, as the effective population size is expected to fluctuate over time, it is likely to further have an impact on the evolutionary dynamics of the non-antigenic gene segment. For example, in periods immediately after a novel antigenic variant has emerged, the effective population size will be extremely low, and thus reassortment is unlikely to confer significant fitness gains in the non-antigenic gene segment. Conversely, if there is sufficient fitness variation in the non-antigenic gene segment(s), then reassortment could enable novel antigenic variants to explore more favorable genetic backgrounds, and out-compete contemporaneous antigenic variants. Although we do not think that our general conclusions will change based on how we exactly model the fitness effects of antigenic mutations, further work needs to be done to unequivocally demonstrate this.

We also did not explicitly consider epistasis in our simulation model. There is evidence that epistatic interactions both within and between gene segments can drive the adaptive evolution of seasonal influenza A viruses. For example, T-cell immune escape mutations in NP have been enabled by stability-mediated epistasis (Gong et al. 2013, 2014) and functional mismatches between the activities of the HA and the NA are known to decrease viral fitness considerably (Wagner et al. 2002; Neverov et al. 2015). However, to effectively model epistasis, a detailed knowledge about the fitness landscape of the virus genome, which is currently lacking, is necessary. Elucidating the epistatic interactions in influenza A viruses should be a focus of future work, since it could help explain the role that intrasubtypic reassortment plays in contributing to the adaptive evolution of seasonal influenza (Neverov et al. 2014), and more broadly, it could help us understand the epidemic (and even pandemic) potential of reassortant viruses.

Our findings that selection is likely to act upon both antigenic and non-antigenic gene segments and that reassortment can influence the rate of virus adaptive evolution have important implications for predicting future influenza strains. In particular, our study indicates that viral mutations are subjected to linkage effects within and to a somewhat lesser extent between gene segments, consistent with the conclusions of Koelle and Rasmussen 2015. As a consequence, we anticipate better forecasting can be achieved if the virus genetic background is considered as a whole, and is not just restricted to HA. This will be largely dependent on obtaining a more comprehensive understanding of the phenotypic variation in other gene segments, which we recommend should be a priority for future research.

## Supplementary Material

Supplementary Information

Supplementary Figure 1

Supplementary Figure 2

Supplementary Figure 3

Supplementary Figure 4

Supplementary Figure 5

Supplementary Figure 6

Supplementary Figure 7

Supplementary Figure 8

Supplementary Figure 9

Supplementary Figure 10
