# Supplementary material for: Selection on non-antigenic gene segments of seasonal influenza A virus and its impact on adaptive evolution
Source: Virus Evol. 2017 Nov 9;3(2):vex034. doi: 10.1093/ve/vex034 (PMC5724400; doi:10.1093/ve/vex034)
Supplement: Supplementary Information [file vex034_supp_info.docx]

**SUPPLEMENTARY FIGURE LEGENDS**

**Figure S1:** Maximum clade credibility (MCC) trees for influenza A/H3N2’s eight gene segments as inferred from the whole genome sequences provided in Bhatt et al. (2011).

**Figure S2:** The difference in the TMRCA of the antigenic and non-antigenic gene segments for (A) model simulations with selection on only the antigenic gene segment, and (B) model simulations with selection on both gene segments. For each model, 500 simulations were run for 60 years. TMRCA differences were calculated for each simulation annually over the last 20 years of the simulation. The 20 calculated TMRCA differences from each simulation were combined over the 500 simulations to produce the shown histograms.

**Figure S3:** The TMRCA dynamics of each of the six internal gene segments, alongside the TMRCA dynamics of the HA (orange).

**Figure S4:** A statistical comparison between TMRCA differences observed empirically versus those obtained via model simulation. For each internal gene segment of influenza A/H3N2, we calculated the set of TMRCA differences between the HA and that internal gene segment, using the MCC trees shown in Figure S1 annually. We then simulated the model with selection occurring on both gene segments 500 times, and simulated the model with selection occurring on only the antigenic gene segment 500 times. Both sets of models were simulated for 60 years. The last 20 years of each of the simulations were used to calculate the TMRCA differences between the antigenic gene segment and the non-antigenic gene segment, again on an annual basis. Each set of simulation-derived TMRCA differences was compared against the set of TMRCA differences that was calculated from the MCC trees using a two-sample Kolmogorov-Smirnov test. Each comparison of TMRCA difference distributions yielded a test statistic *D*, which quantifies the maximum distance between the two cumulative distribution functions. Here, we plot histograms of the *D* test statistic for each of the two models (with and without selection on the non-antigenic gene segment) when compared again the empirical TMRCA differences. Each panel takes the internal (non-antigenic) gene segment of influenza A/H3N2 to be a different gene segment (PB2, PB1, PA, NP, MP, NS). The dashed vertical lines show the value of the *D* statistic at which one can reject the null hypothesis that the two compared sample distributions come from the same underlying distribution (at a significance level of *α* = 0.05). The shaded regions correspond to where the null hypothesis is rejected. The results show that the model with selection occurring on both gene segments more frequently generates TMRCA differences between the antigenic and non-antigenic gene segments that cannot be said to come from a different underlying distribution than the one that generated the TMRCA differences calculated from the empirical flu data.

**Figure S5:** Distribution of TMRCAs of antigenic (red) and non-antigenic (blue) gene segments under different infected population sizes (*N* = 1000, 5000, and 10000). These results are based on 100 simulations. Selection on both gene segments is assumed, and the rate of coinfection is *β* = 0.0125 per day.

**Figure S6:** Distribution of TMRCAs of antigenic (red) and non-antigenic (blue) gene segments under three different mutation rates: *U *= 0.05, 0.1, and 0.2 per genome per transmission event. As in Figure S5, these distributions were based on 100 simulations. Selection on both gene segments is assumed, the rate of coinfection is *β* = 0.0125 per day, and the infected population size is *N* = 1000.

**Figure S7:** Distribution of TMRCAs of antigenic (red) and non-antigenic (blue) gene segments at different rates of coinfection corresponding to *β* = 0.0025, 0.0125, and 0.25 per day. These distributions are based on 500 simulations. Selection on both gene segments is assumed, and the infected population size is *N* = 1000.

**Figure S8:** Cumulative distribution of fitness effects quantified by Visher et al (2016) for HA and NA (orange) and the internal gene segments (blue).

**Figure S9: Adaptive evolution of the virus at varying levels of coinfection** **when both gene segments undergo selection,** using an alternative distribution of fitness effects based on Visher et al (2016). The mean (log) population fitness are shown for (A) the whole virus, (B) the antigenic gene segment, and (C) the non-antigenic gene segment for three different coinfection rates (*β* = 0, *β* = 0.0125, and *β* = 0.025 per day). 500 simulations were run at each of the three coinfection levels. Solid lines show the means of the simulations’ mean (log) population fitness levels.

**Figure S10:** The rate of viral adaptive evolution and gene segment-specific patterns of genetic diversity under a model where both gene segments experience the same amount of selection and at three different coinfection rates (*β* = 0, *β* = 0.0125, and *β* = 0.025 per day).. (A) The mean (log) population fitness of the whole virus over time. (B) The distribution of TMRCAs for each of the two gene segments under the three different coinfection rates.

**SUPPLEMENTARTY INFORMATION**

**Text S1: Calculation of coinfection level:**

The equilibrium number of singly-infected individuals *I*_s_ and the equilibrium number of coinfected individuals *I*_co_ can be easily calculated given the infection/recovery rate *α* and the coinfection rate *β*. To do this, we note that the overall rate at which individuals become coinfected is given by *βI*_s_, which is equivalent to *β* (*N*- *I*_co_), since we assume a constant number *N* of infected individuals in the population. The overall rate at which coinfected individuals recover is given by *αI*_co_. The number of individuals who are coinfected is at equilibrium when the rate at which they arise is equal to the rate at which they are removed: *β* (*N*- *I*_co_) = *αI*_co_. Solving this for *I*_co_ yields $I_{co}=\frac{\beta}{(\alpha+\beta)}N$, such that the equilibrium fraction of the population that is coinfected is given by: $\frac{I_{co}}{N}=\frac{\beta}{(\alpha+\beta)}$.
