# Supplementary figures and images for: Selection on non-antigenic gene segments of seasonal influenza A virus and its impact on adaptive evolution
Source: Virus Evol. 2017 Nov 9;3(2):vex034. doi: 10.1093/ve/vex034 (PMC5724400; doi:10.1093/ve/vex034)

**HA**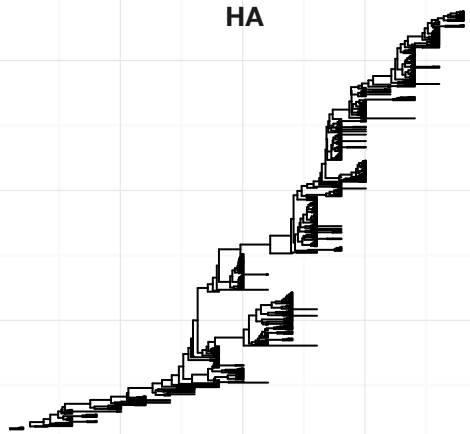**PB2**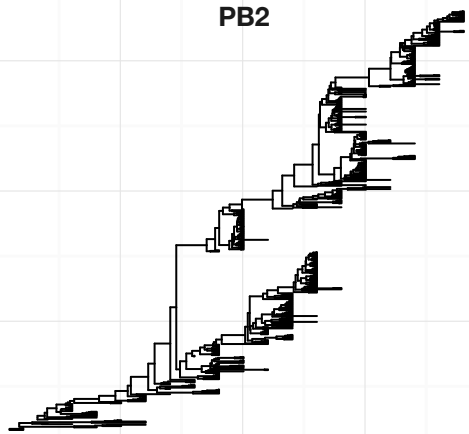**PB1**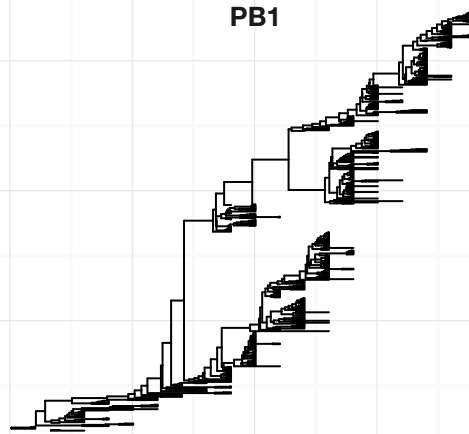**PA**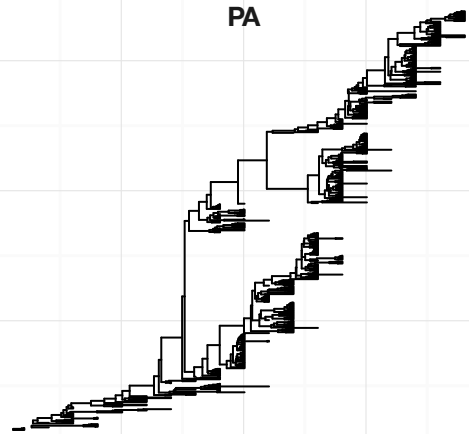**NA**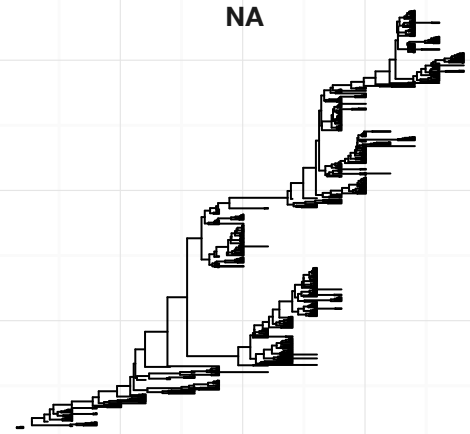**NP**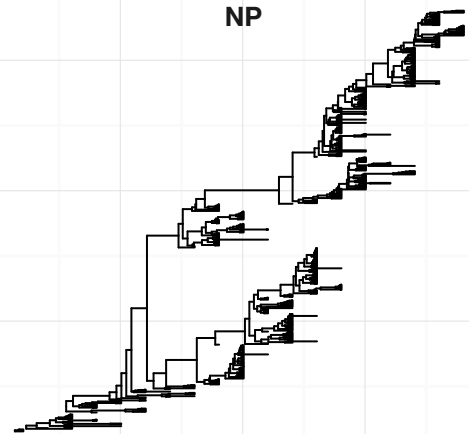**M1**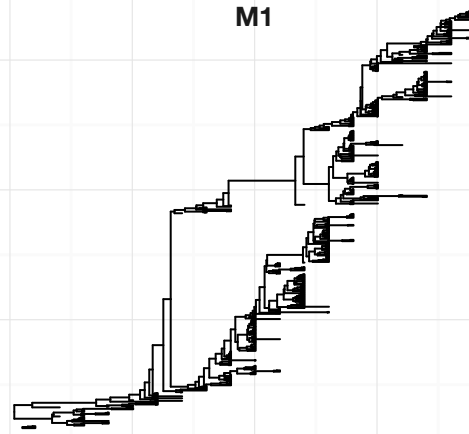**NS**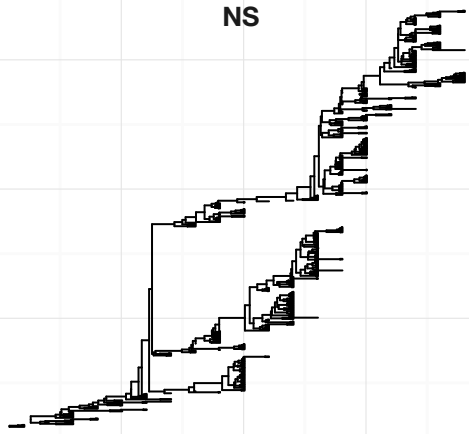

Time (years)

Supplement: Supplementary Figure 1 [file vex034_supp_figure_s1.pdf]

Selection on only the antigenic  
gene segment

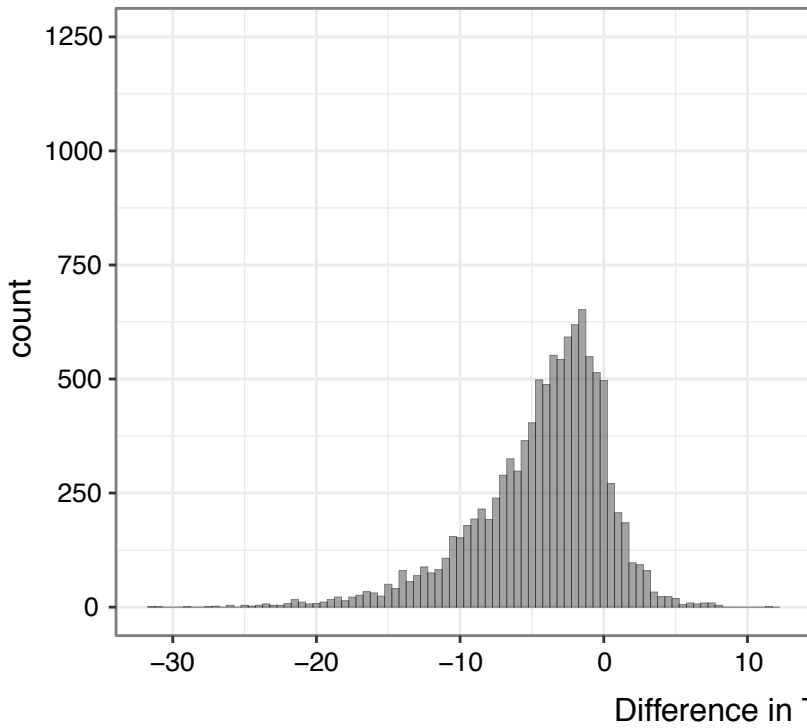

Selection on both gene  
segments

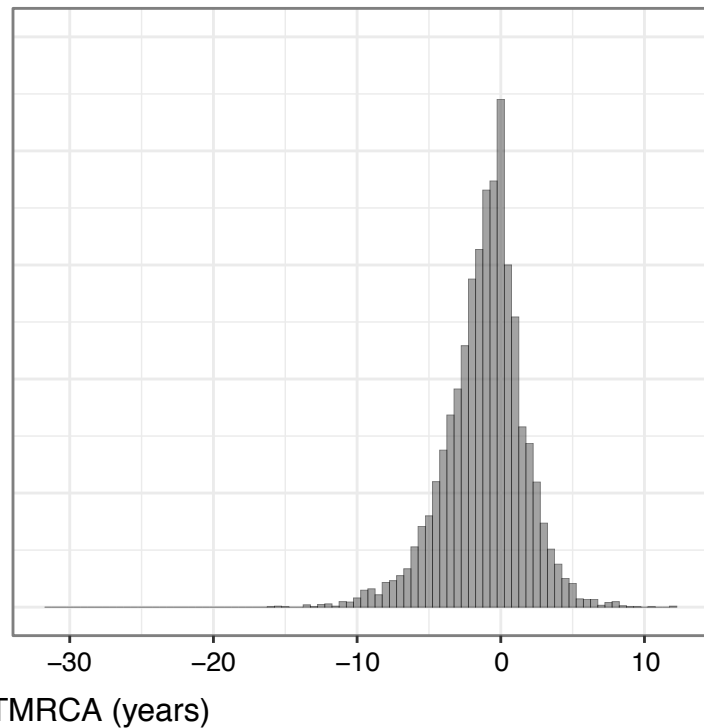

Supplement: Supplementary Figure 2 [file vex034_supp_figure_s2.pdf]

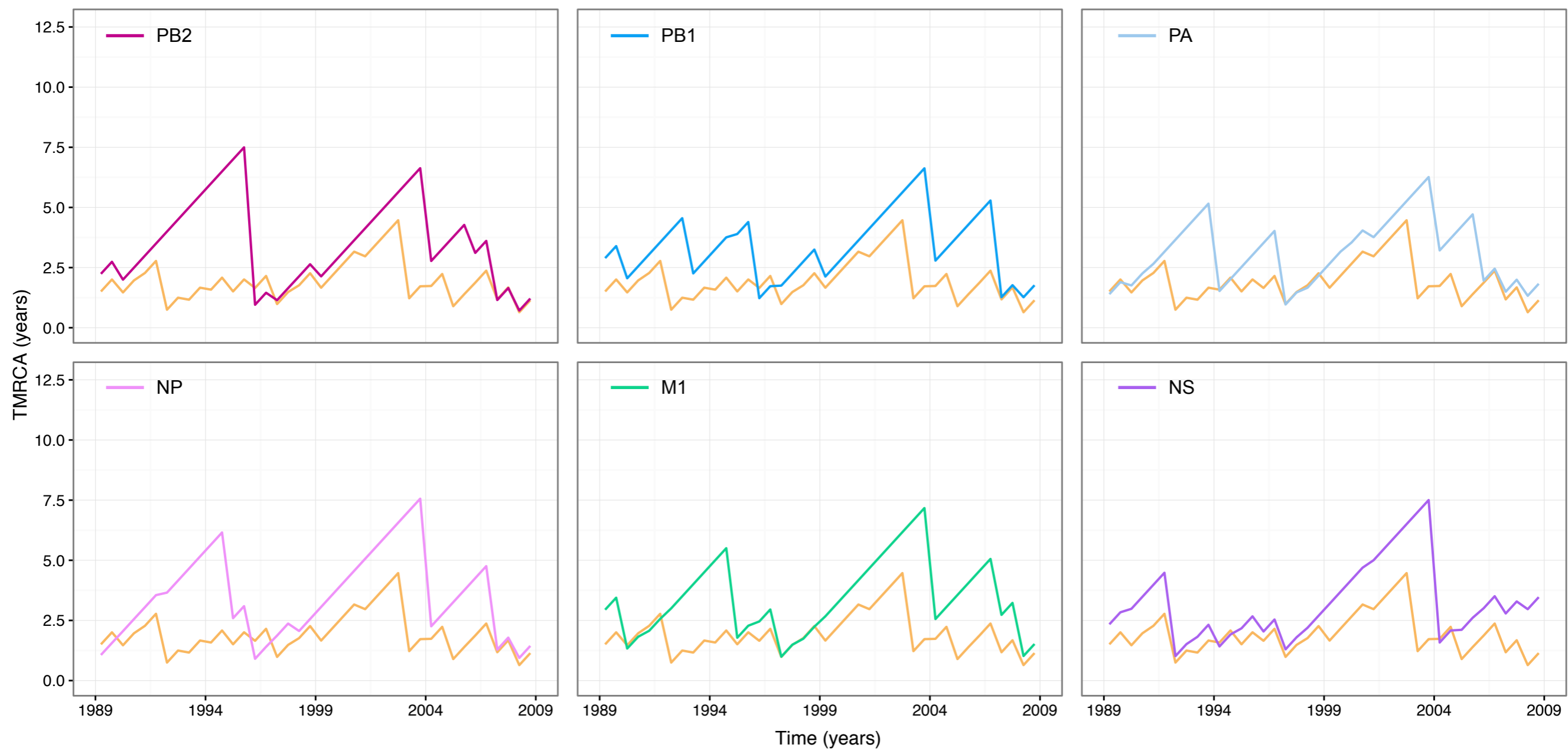

Supplement: Supplementary Figure 3 [file vex034_supp_figure_s3.pdf]

Selection on only the  
antigenic gene segment

Selection on both  
gene segments

**PB2**

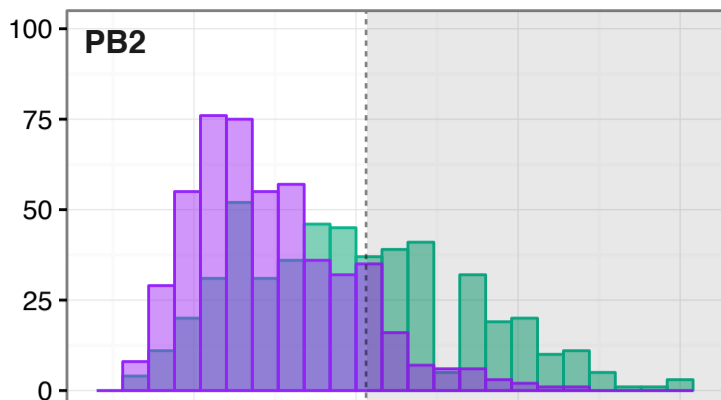

**PB1**

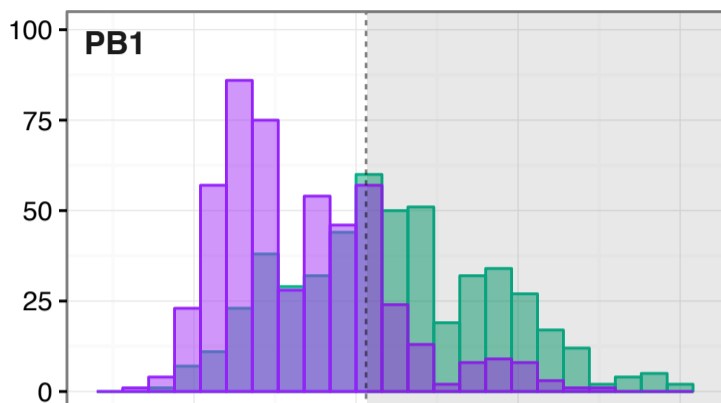

**PA**

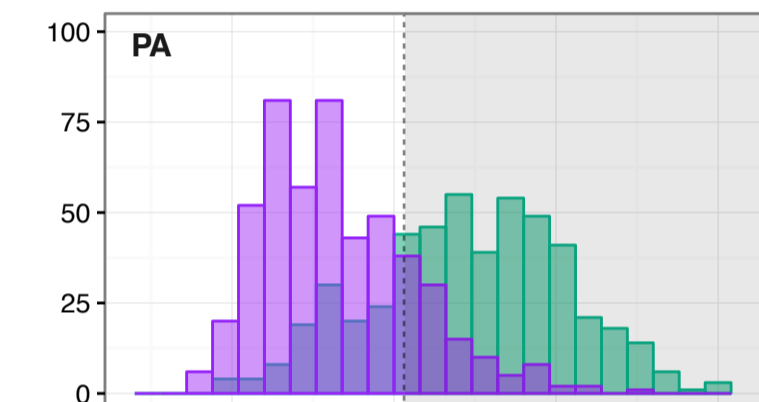

**NP**

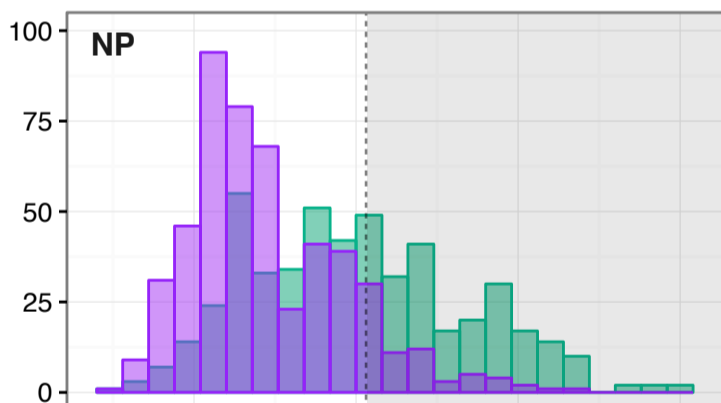

**MP**

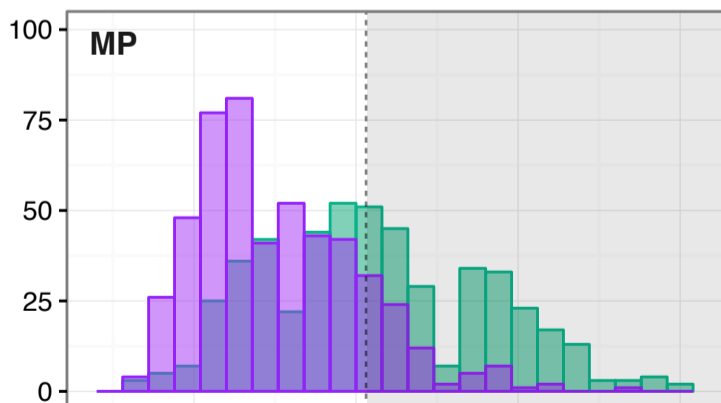

**NS**

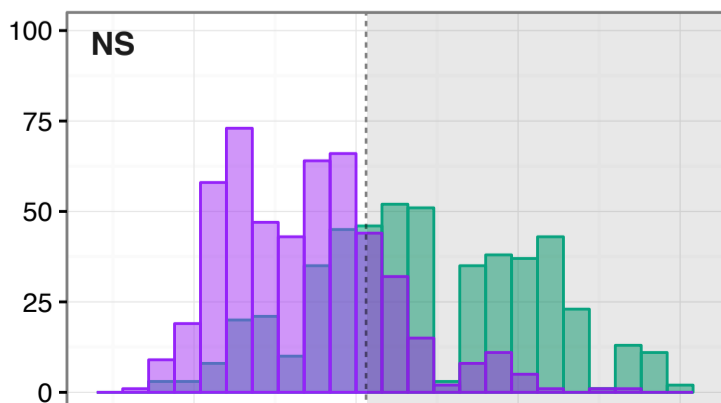

Supplement: Supplementary Figure 4 [file vex034_supp_figure_s4.pdf]

$N = 1000$

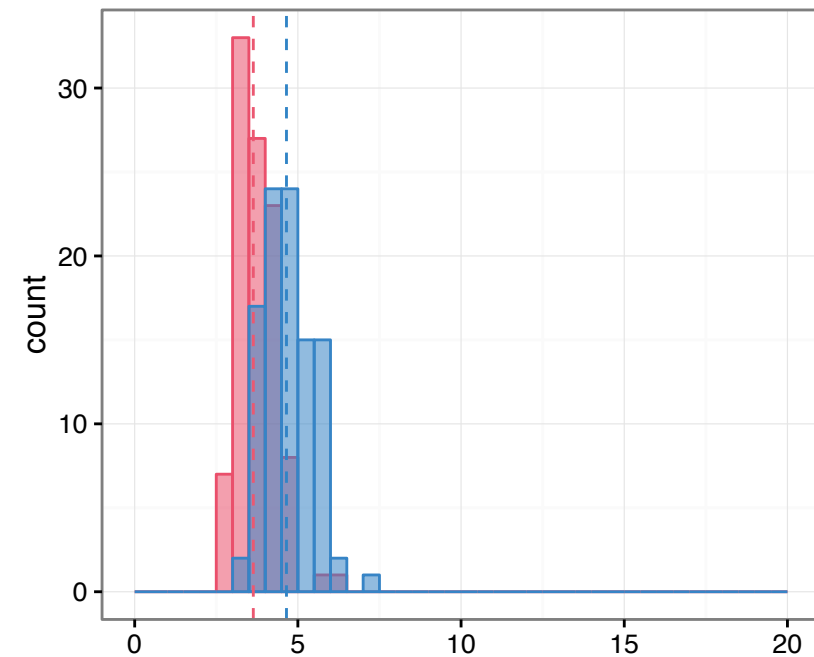

$N = 5000$

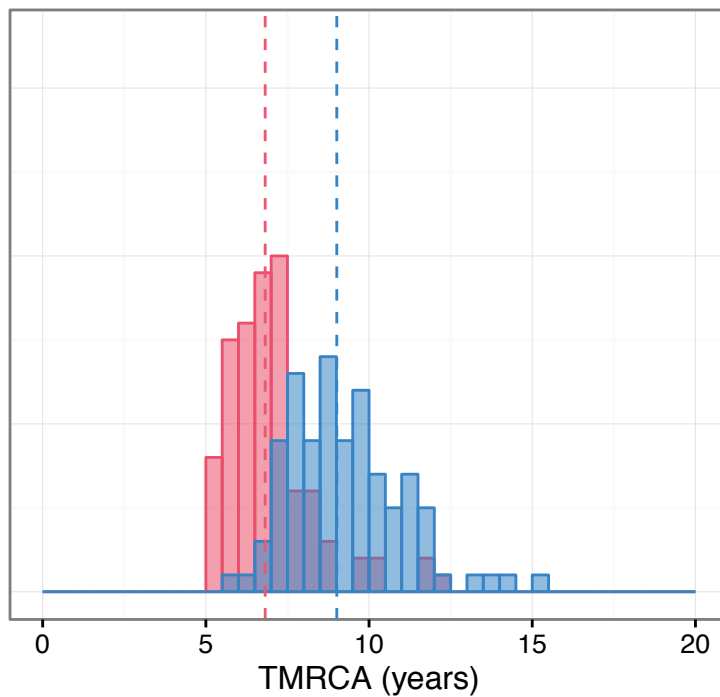

$N = 10000$

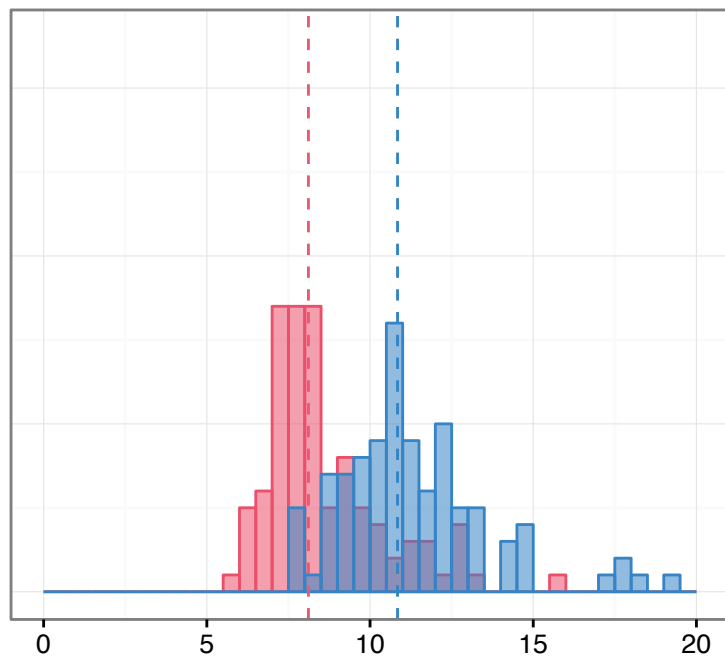

Supplement: Supplementary Figure 5 [file vex034_supp_figure_s5.pdf]

$U = 0.05$ 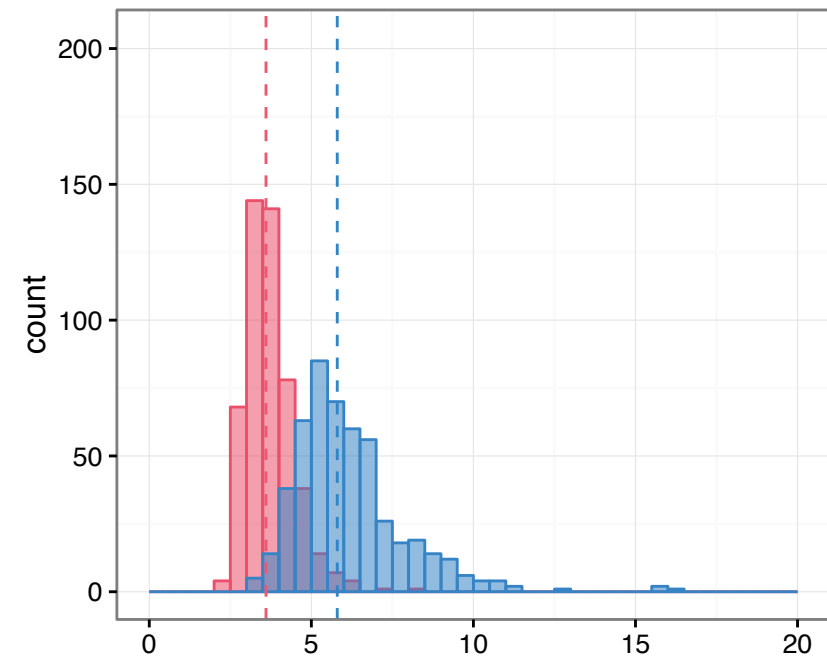 $U = 0.1$ 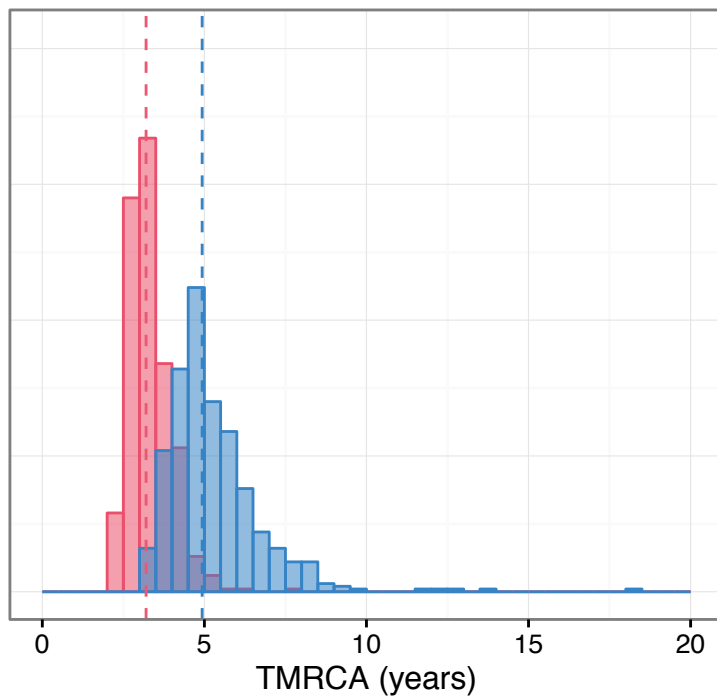 $U = 0.2$ 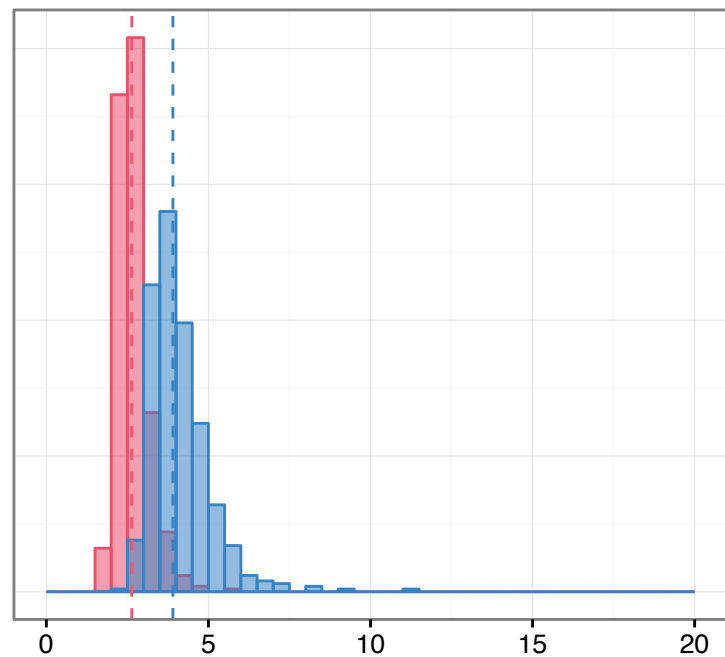

Supplement: Supplementary Figure 6 [file vex034_supp_figure_s6.pdf]

increasing coinfection rate

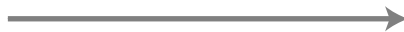

$\beta = 0.0025$

$\beta = 0.0125$

$\beta = 0.25$

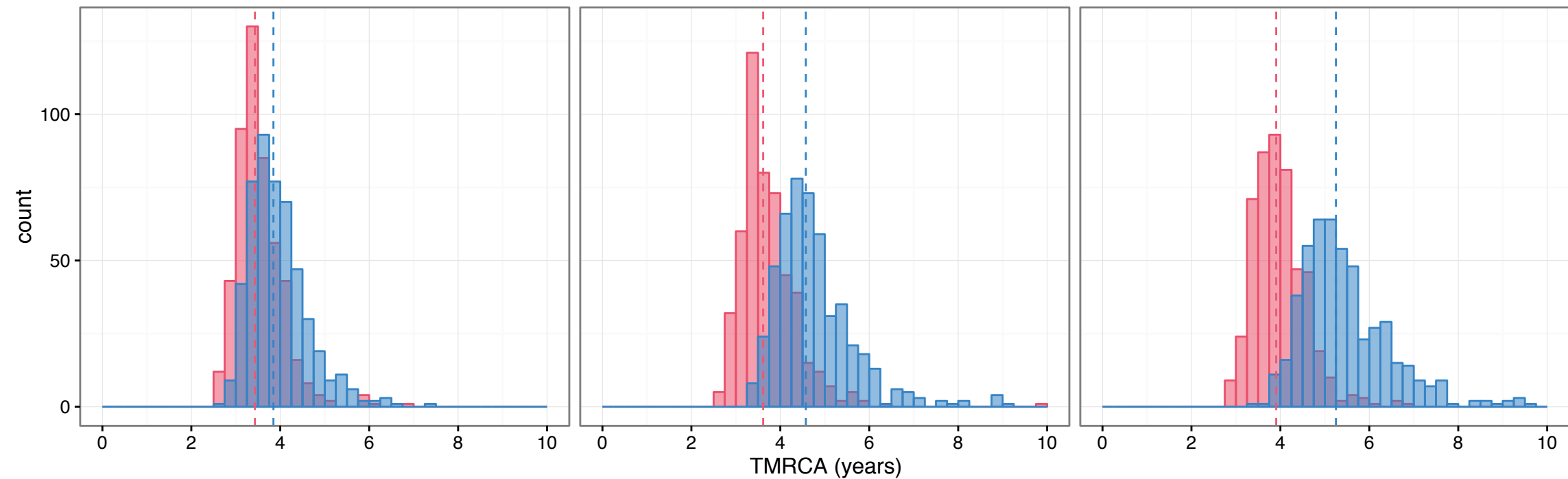

Supplement: Supplementary Figure 7 [file vex034_supp_figure_s7.pdf]

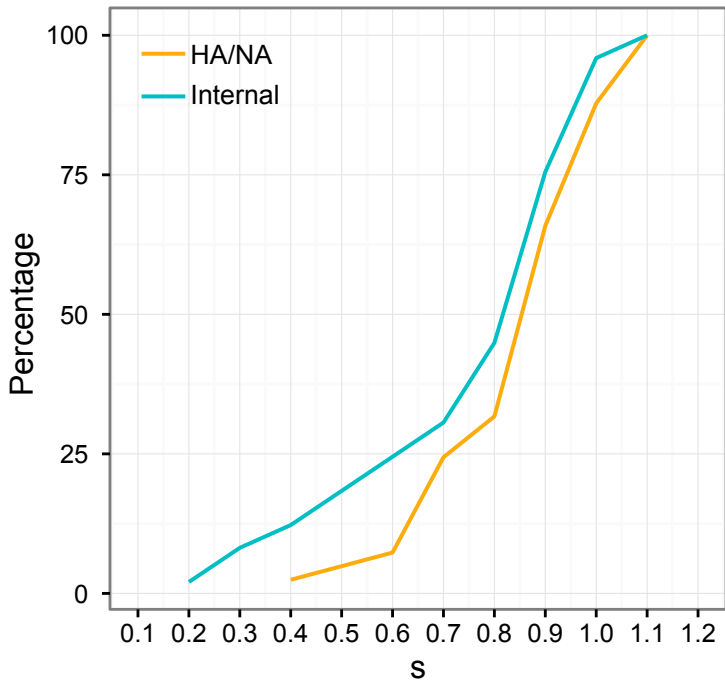

Supplement: Supplementary Figure 8 [file vex034_supp_figure_s8.pdf]

# **A** Whole Virus

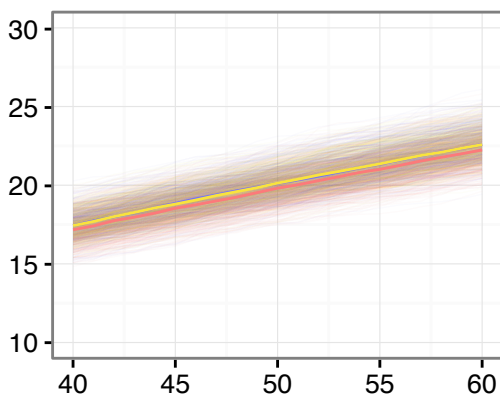

# **B** Antigenic Segment

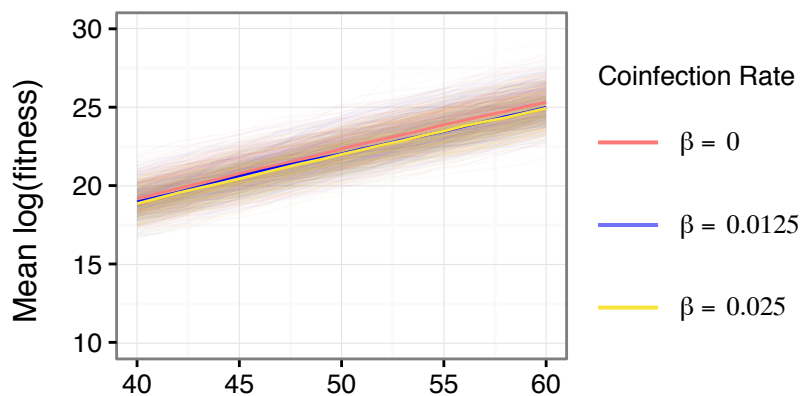

# **C** Non-antigenic Segment

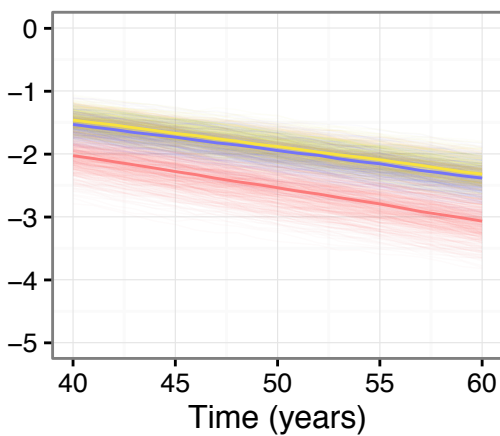

Supplement: Supplementary Figure 9 [file vex034_supp_figure_s9.pdf]

**A**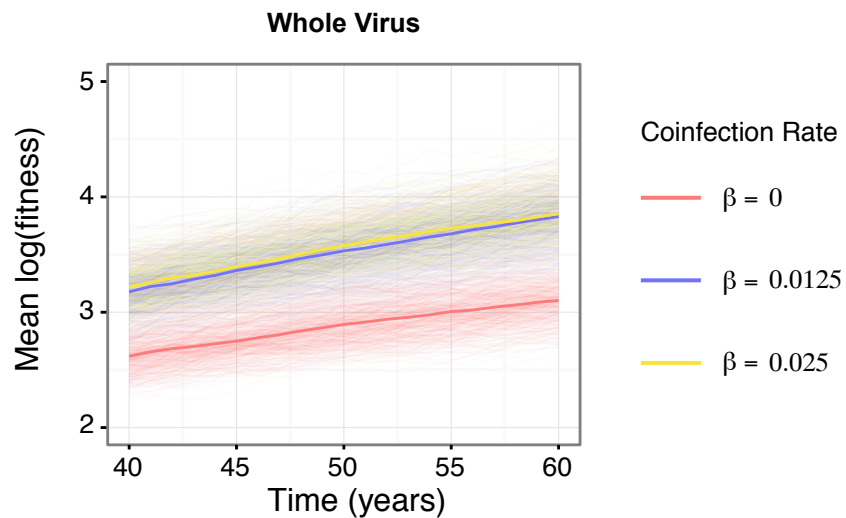**B**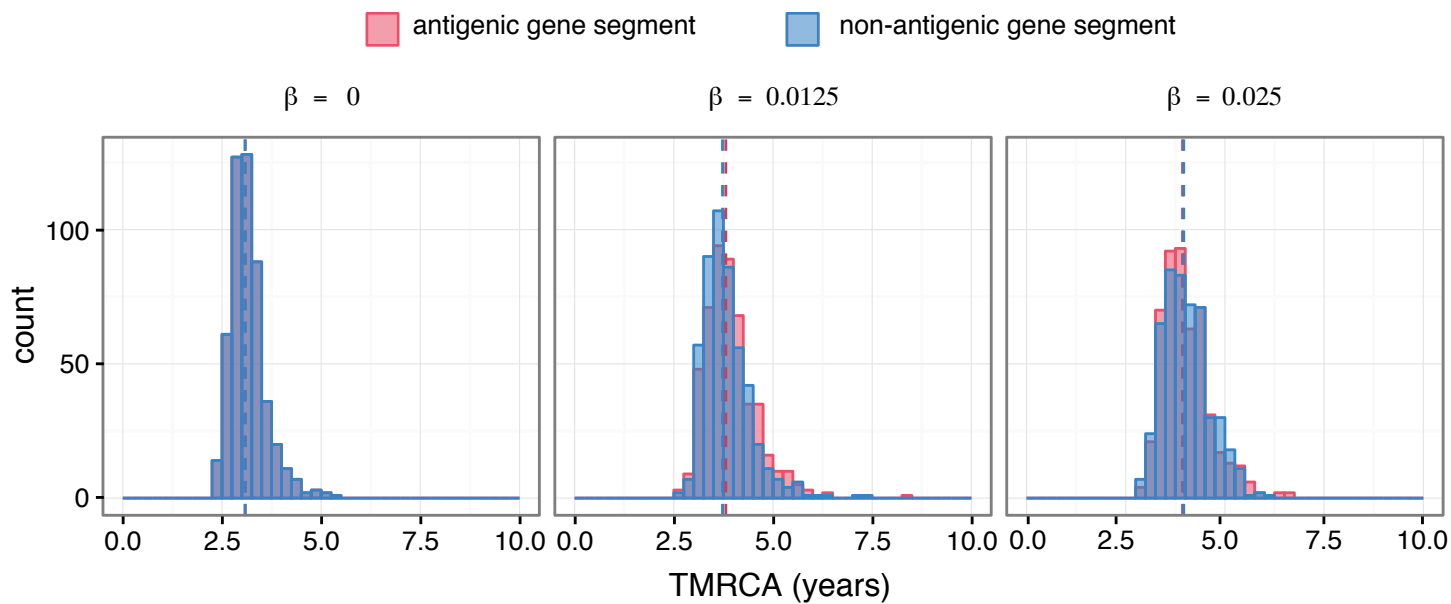

Supplement: Supplementary Figure 10 [file vex034_supp_figure_s10.pdf]
